# Predicting high-level visual areas in the absence of task fMRI

**DOI:** 10.1038/s41598-024-62098-9

**Published:** 2024-05-18

**Authors:** M. Fiona Molloy, Zeynep M. Saygin, David E. Osher

**Affiliations:** 1 Department of Psychology, The Ohio State University, 1835 Neil Avenue, Columbus, OH 43210 USA; 2 Department of Psychiatry, University of Michigan, Ann Arbor, MI USA

**Keywords:** Functional connectivity, High-level vision, Functional regions of interest, fMRI, Perception, Visual system, Machine learning, Functional magnetic resonance imaging

## Abstract

The ventral visual stream is organized into units, or functional regions of interest (fROIs), specialized for processing high-level visual categories. Task-based fMRI scans (“localizers”) are typically used to identify each individual’s nuanced set of fROIs. The unique landscape of an individual’s functional activation may rely in large part on their specialized connectivity patterns; recent studies corroborate this by showing that connectivity can predict individual differences in neural responses. We focus on the ventral visual stream and ask: how well can an individual’s resting state functional connectivity localize their fROIs for face, body, scene, and object perception? And are the neural processors for any particular visual category better predicted by connectivity than others, suggesting a tighter mechanistic relationship between connectivity and function? We found, among 18 fROIs predicted from connectivity for each subject, all but one were selective for their preferred visual category. Defining an individual’s fROIs based on their connectivity patterns yielded regions that were more selective than regions identified from previous studies or atlases in nearly all cases. Overall, we found that in the absence of a domain-specific localizer task, a 10-min resting state scan can be reliably used for defining these fROIs.

## Introduction

A major contribution of human brain mapping is the discovery of the organization of the ventral visual stream into category-selective units of visual processing^1,2^. Advances in non-invasive neuroimaging, specifically functional magnetic resonance imaging (fMRI), have revealed regions in the inferior temporal cortex that are specialized for specific visual categories, such as faces (e.g., in the FFA, or fusiform face area^3^), bodies (e.g., in the EBA, or extrastriate body area^4^), and scenes (e.g., in the PPA, or parahippocampal place area^5^). Other studies have revealed the presence of additional specialized regions, such as a region specialized for orthography (visual word form area, VWFA^6^), and, more recently, food-processing units^7^.These regions, called functional modules or functional regions of interest (fROIs), also exist in domains outside of vision, such as language and theory of mind^8^.

These fROIs can be identified within an individual using task-based fMRI localizers^9–11^. fROIs are not homogenous across individuals, as individual differences exist in the location, size, and shape of these fROIs^12^. Identifying these regions is important for researchers who want to investigate how they function. Further, exploring how category-selective regions vary across individuals and how they relate to behavior can help reveal the mechanisms that govern visual selectivity. Using fROIs also has practical advantages, such as providing a standard brain space circumventing difficulties registering across subjects, and limiting the number of comparisons thus lowering the false positive rates^13^.

The gold standard for identifying fROIs is using an independent task-based localizer. For visual regions, these localizer tasks often involve viewing pictures or videos of different visual stimuli, running a generalized linear model to compute activation for each category (often through contrasting a different category), and then defining the fROI either by hand-selecting active voxels, or by using a user-specified threshold within a prespecified region (e.g., the group-constrained subject specific approach^9,11^). Note this localizer run must be done independently to avoid circularity. Often, this necessitates running many additional task runs to localize regions which may limit data collection for the experiment of interest.

In the absence of localizer task data, researchers can identify fROIs using group-level atlases. These atlases could be a domain-general whole-brain parcellation, constructed from cytoarchitecture (such as Brodmann areas^14,15^), structural MRI^16,17^, or multiple modalities, including task data (e.g., the Glasser multi-modal parcellation^18^). These group-level atlases could alternatively be comprised of probabilistic parcels constructed for a specific domain (such as language or high-level vision^11,19^). In addition to saving scanner time, these atlases are usually easy to apply to data and provide a common ground for comparisons across studies and populations. Further, these approaches allow for secondary data analyses of existing datasets that did not collect a localizer task in a domain of interest. However, these atlases do not account for individual differences, which are known to be extensive in fROIs.

There may be a third way to identify fROIs through an individual’s own neural circuitry. Leveraging connectivity data (either structural connectivity from diffusion weighted imaging or functional connectivity from resting state fMRI) could provide an alternative to atlas-based approaches. This can be useful in analyzing data that have already been collected that did not include an appropriate localizer task (or even any task data). In particular, many of the large open-access neuroimaging datasets included limited or no runs of a high-level visual task, but collected extensive connectivity data^20^. Additionally, there exist large and stable individual differences in connectivity^21^, and connectivity relates to behavior and cognition^22^. Because of this, connectivity could relate to individual differences in fROIs as well. Saygin et al.^23^ demonstrated that an individual’s structural connectivity patterns can predict that individual’s face activity in the fusiform gyrus. Later, Osher et al.^24^ generalized these findings, using structural connectivity to predict category-responses to faces, bodies, scenes, and objects in additional regions. Together, these studies indicate the promise of using an individual’s connectivity to map individual-specific ventral visual areas. However, they predict the degree of responsiveness of individual voxels, as opposed to identifying fROIs directly. Further, both approaches used relatively coarse anatomical parcels, such as the entire fusiform gyrus, as a spatial constraint of individual models. Here, we use resting state functional connectivity, and more precise search spaces^11^, in order to identify 18 regions that selectively respond to faces, bodies, scenes, or objects within single subjects.

In addition to these structural connectivity predictions, previous research has demonstrated a close link between *functional* connectivity and task activity^25^. Note that while structural and functional connectivity are correlated, the link between these two measures remains unclear^26^. Using resting state functional connectivity from the Human Connectome Project^27^, Cole et al.^28^ and Tavor et al.^29^ predicted task activity and contrast maps, respectively, for seven different tasks, including working memory, gambling, motor, language, social relational, and emotion. Since then, resting state functional connectivity has been shown to be predictive of an individual’s task activity in other domains, such as in auditory and visual selective attention areas in the frontal lobe^30^, in the frontoparietal network^31^, and in the language network^32^. Most recently, researchers have proposed improvements to the model presented in Tavor et al.^29^ using more complex machine learning methods^33–35^.

Here, we test a potential application of functional connectivity predictive modeling by exploring the feasibility of using these model predictions *to define fROIs* without using any task data, in the absence of functional localizers. We compute these regions within parameters that closely represent the practicalities of a standard experiment. Instead of using 60 min of resting state data (as was done in ^28,29,33–35^), we use a single resting state scan of approximately 10 min. The performance of these connectivity-predicted fROIs will be evaluated based on if they are indeed selective for their preferred category and outperform other (e.g., atlas) approaches.

First, we describe the nested cross-validation procedure to construct and fit the predictive connectivity models for fROIs selective for objects, scenes, faces, and bodies from 40 adults with a 10-min resting state session and two runs of a dynamic localizer task. Second, we demonstrate the selectivity of the resulting fROIs based on data from the left-out task run. Third, we ask whether the connectivity-predicted fROIs outperform group-level atlas approaches. Finally, we investigate regional and hemispheric differences in the selectivity of connectivity-predicted fROIs. Overall, we find resting state functional connectivity can generate individual-specific regions that are selective for high-level visual categories. We conclude by discussing the variable nature of the structure–function relationship, and how it may vary throughout the brain or generalize to other cognitive domains and populations.

## Methods

**Figure 1:**
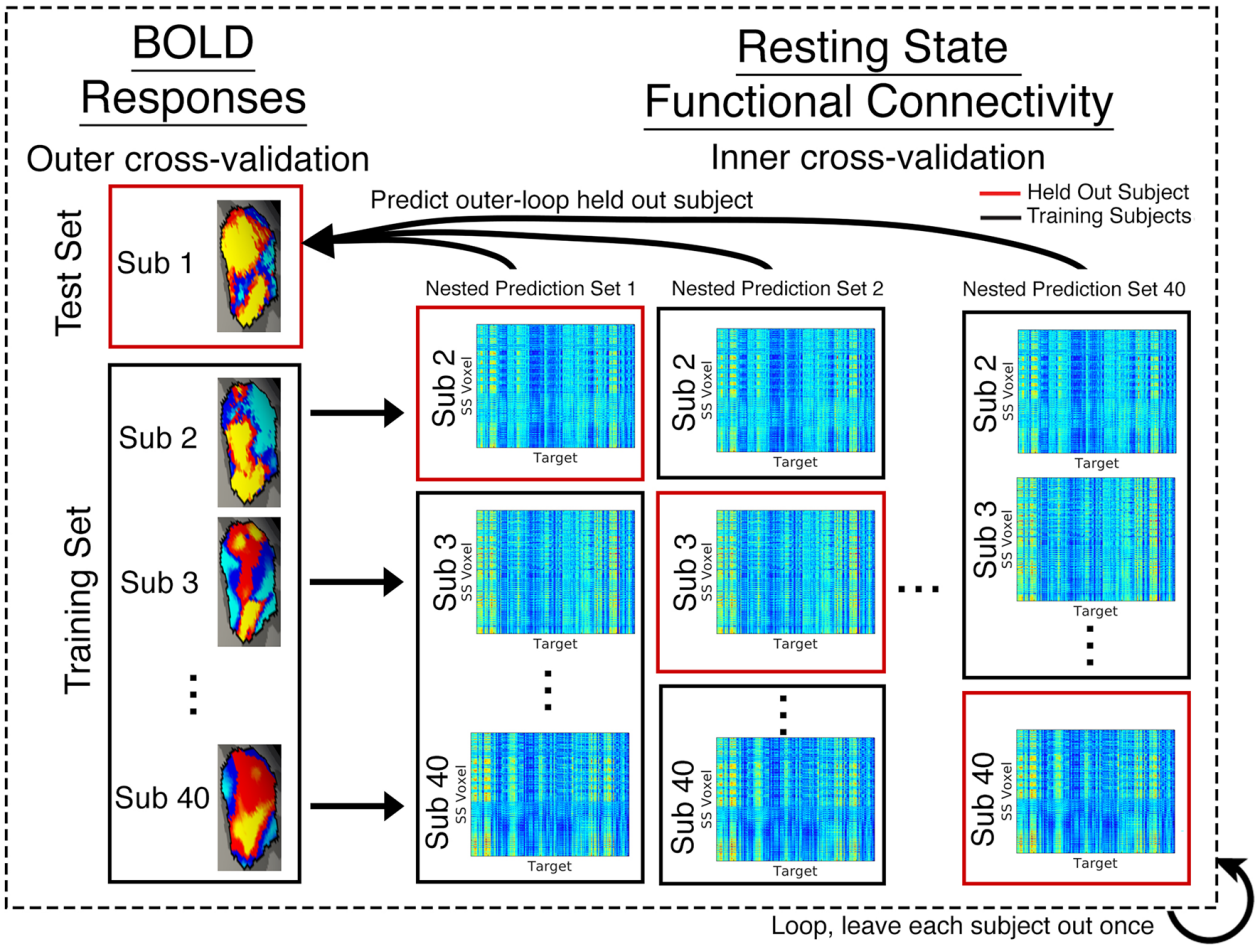
Model fitting schematic. For each fROI (right FFA pictured as an example), we trained a model to predict an individual’s task activity (each search space^11^ voxel’s response to the preferred category) using that same individual’s functional connectivity at rest (each search space voxel’s connectivity to the entire brain). A nested cross validation loop was constructed to optimize the model’s parameters (e.g., λ for ridge regression models) without overfitting the model.

An overview of the predictive modeling approach is summarized in Fig. 1. Briefly, we predict an individual’s task activation to a specific category within a putative ventral visual fROI^11^ using that individual’s resting state connectivity within that region to the rest of the brain. The connectivity-predicted fROIs are then defined from those model predictions. A nested cross-validation approach is used to train and optimize the model and resulting fROIs are evaluated based on expected categorical selectivity and individual specificity.

### Participants

The sample included 40 participants (21 females and 19 males) between the ages of 18 and 55 (mean = 24.63 years, standard deviation = 6.74 years) recruited at The Ohio State University. The study was approved by the Institutional Review Board at The Ohio State University, informed consent was obtained from all participants, and the study was conducted following all relevant guidelines and regulations.

### Acquisition

Each participant completed two runs of a functional localizer task, one resting state run, and a high-resolution structural scan. The task was a dynamic visual localizer^36^ with short video clips of faces, bodies, scenes, objects, and scrambled objects (TR = 1000ms, TE = 28ms, voxel size = 2 × 2 × 3mm). Participants were asked to passively view 3-s video clips of each category. The face and body conditions consisted of video clips of young children’s faces and body parts (e.g., hands, legs) respectively naturally moving with a black background. The object condition consisted of different moving objects (toys) filmed on a black background (e.g., blocks swinging), and scrambled objects were constructed by scrambling each frame of the object videos into a 15 × 15 grid. For each category, six 3-s video clips were shown per block (18 s per condition). One run (total TR = 234, 3.9 min total) contained 2 blocks per condition, with 3 rest blocks (6 s of alternating full screen colors) at the beginning, middle, and end of each run. The order of conditions for each participant was randomized.

A 9.67-min resting state scan (TR = 1000ms, TE = 28ms, voxel size = 2 × 2x3mm) was also acquired for all participants (total TR = 580). A white fixation cross was presented on a black background. Participants were instructed to keep their eyes open, look at the fixation cross, and think of nothing in particular.

### Preprocessing

All structural images were preprocessed using FreeSurfer’s recon-all pipeline with default parameter values (https://surfer.nmr.mgh.harvard.edu/fswiki/recon-all/). Briefly, this includes intensity correction, skull stripping, surface co-registration, spatial smoothing, white matter and subcortical segmentation, and cortical parcellation.

Task data were motion-corrected and time points with more than 1mm total vector movement between TRs were identified and regressed out as framewise nuisance regressors in the task data analyses. These data were then distortion corrected, registered to anatomical space using bbregister^37^, and resampled to the T1 weighted anatomical image. Finally, data were detrended and spatially smoothed with a 4mm FWHM kernel.

Resting state data were preprocessed using FS-Fast (https://surfer.nmr.mgh. harvard.edu/fswiki/FsFastAnlysisBySteps) to complete motion correction and to smooth gray matter with a 3mm FWHM kernel. We also censored datapoints with framewise displacement greater than 0.5 mm.

### Defining the connectome

Matrices of functional connectivity were defined for each high-level visual fROI search space from Julian et al.^11^. In Julian et al., the search spaces were constructed from the task fMRI data of 30 participants who completed 4 runs of the dynamic localizer task also used in the present study. We included 9 bilateral fROIs (18 regions total): 3 face regions (FFA: fusiform face area, OFA: occipital face area, STS: superior temporal sulcus), 1 body region (EBA: extrastriate body area), 3 scene regions (PPA: parahippocampal place area, RSC: retrosplenial cortex, TOS: transverse occipital sulcus), and 2 object regions (LO: lateral occipital, PFS: posterior fusiform sulcus). These search spaces were projected to the fsaverage template surface and the resulting search spaces are shown in Supplementary Figure S1. The connectome for a given fROI was defined as the Fisher-transformed Pearson product correlation between the timeseries of each vertex in the fROI search space and the averaged timeseries within each Glasser multimodal parcel on that same hemisphere (179 total). Thus, the dimensions of the fROI connectome were then the number of vertices in the search space × 179.

### Computing task activity

The preprocessed task data were analyzed using a first-level GLM in individual subject volume space. Regressors were specified for each visual category (faces, bodies, scenes, objects, and scrambled objects), as well as 6 motion nuisance regressors. We specified a standard boxcar function (block design) and a canonical hemodynamic response function (standard gamma function with d = 2.25 and t = 1.25). Contrasts were defined as: Faces > Objects, Bodies > Objects, Scenes > Objects and Objects > Scrambled Objects. These contrasts are commonly used to localize face-, body-, scene-, and object-selective regions, respectively. The resulting contrast significance maps and β estimates were projected into fsaverage space and used for the following modeling and analysis.

### Predictive model training

We designed ℓ_2_ regularized linear models to predict neural activity measured from the dynamic localizer task, using resting state functional connectivity as regressors, for each of the 18 search spaces. Functional connectivity of each vertex to 179 ipsilateral cortical regions, as well as curvature, thickness, and x, y, and z coordinates of the vertex, comprised the full design matrix. Models were trained using nested cross-validation in order to optimize the regularization hyperparameter λ. Models were fit separately for each fROI. In this framework, we first predicted the category-selective activity of each voxel within a search space for a given individual, based on the functional connectivity of all of their vertices in the search space to the rest of the brain. We trained the model using the data of all but one individual, leaving each subject out once. However, the λ hyperparameter of the trained model must also be determined. To avoid overfitting the model and avoid bias, we used a nested cross-validation approach. Within each training set, we again left each subject out once and optimized the hyperparameter λ (inner cross validation). Potential λ values were defined over a vector of 100 logarithmically spaced values between decades 10^–5^ and 10^2^. We identified the optimal λ that minimized mean squared error of the left-out subjects in the inner cross validation loop, and then applied this model to the left-out subject in the outer cross-validation loop. We repeated this process leaving out each outer-loop subject once. A final model trained on all 40 subjects is available online (https://github.com/CognitionBrainCircuitryLab/Predict_fROIs).

### Model evaluation

If connectivity-predicted fROIs may be used in place of task localizers, the predicted fROIs must indeed be selective for their domain of interest. We first defined fROIs for both the actual task contrast maps and predicted contrast maps by selecting the top 10% of vertices within the search space (i.e., Julian parcels) for each fROI. On average, we are predicting 53.88% of the explainable overlap in fROI, and found the predicted fROIs performed about the same across different thresholds (defining fROIs from the top 20% and top 30%) when accounting for chance (Supplementary Figure S2). The predicted fROI was defined from the connectivity model predictions, and the actual fROI was defined using the first run of the localizer task to ensure independence between voxel selection and measures of selectivity. Predicted fROIs for an example individual are shown in Supplementary Figure S3. We assessed whether each fROI was significantly selective for its respective visual category by performing a paired *t*-test between the percent signal change (PSC) for its respective visual category vs. the PSC of the contrast category. We similarly quantified selectivity as the difference between an fROI’s PSC for its respective visual category vs. the PSC of the contrast category.

The selectivity of each fROI was then compared to three separate atlases: the search spaces from Julian et al.^11^, the closest parcel(s) from the Glasser et al.^18^ multi-modal parcellation, and the corresponding parcel(s) from the Rosenke et al.^19^ visfAtlas. Glasser parcels that had more than 5% overlap with a given Julian search space were assigned to that fROI. The following fROIs were matched to the corresponding Rosenke parcels: the FFA includes the mFus (mid-lateral fusiform gyrus) and pFus (posterior lateral fusiform gyrus); the OFA includes the bilateral IOG (inferior occipital gyrus); the EBA includes the bilateral ITG (inferior temporal gyrus), MTG (middle temporal gyrus), and LOS (lateral occipital sulcus); the PPA includes the bilateral COS (collateral sulcus); and the TOS includes the rhTOS (right transverse occipital sulcus).

Next, we explored regional differences in selectivity across the connectivity-predicted fROIs. Selectivity values were averaged within each visual category (e.g., faces) to explore differences by category in high-level visual regions. Finally, we investigated hemispheric and category effects on an individual subject level using a three-way analysis of variance (ANOVA) to analyze the effect of hemisphere (left or right) and category (body, face, object, and scene) on the mean selectivity values within the connectivity-predicted fROIs for each subject. Subjects were included as a random variable. Post-hoc paired sample two-sided *t*-tests were calculated to investigate hemispheric differences within each visual category.

## Results

Here, we predict an individual’s fROIs in the ventral visual stream using that individual’s unique pattern of whole-brain resting state functional connectivity. These fROIs include three face regions (FFA: fusiform face area, OFA: occipital face area, STS: superior temporal sulcus), one body region (EBA: extrastriate body area), three scene regions (PPA: parahippocampal place area, RSC: retrosplenial cortex, TOS: transverse occipital sulcus), and two object regions (LO: lateral occipital, PFS: posterior fusiform sulcus). The left hemisphere and right hemisphere fROIs were computed separately for a total of 18 regions. We constructed and validated a predictive model for each region using a sample of 40 individuals who completed an approximately 10-min resting state scan and two runs of a dynamic localizer task.

### Connectivity fROIs exhibit expected category selectivity

**Figure 2:**
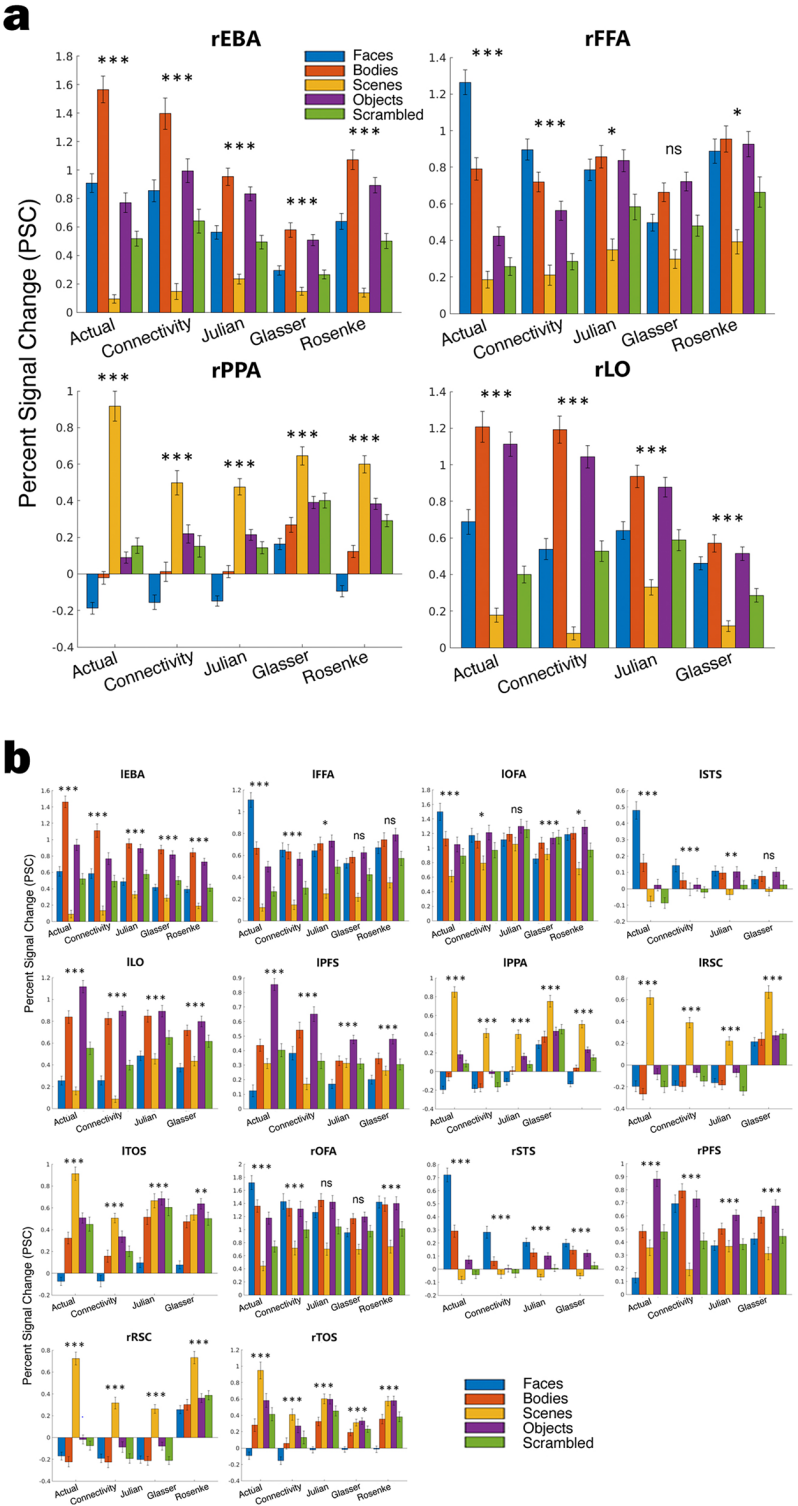
Percent Signal Change (PSC) of each method in each fROI. (**a**) The four panels correspond to four key ROIs: the right EBA (selective for bodies), the right FFA (selective for faces), the right PPA (selective for scenes), and the right LO (selective for objects). Within each plot, the grouped bars correspond to the different approaches for defining fROIs. From left to right, “connectivity” is the fROI predicted from resting state connectivity, “Julian” is from Julian et al. (2012), “Glasser” is from the Glasser et al. (2016) multi-modal parcellation, and “Rosenke” is from the Rosenke et al. (2020) visfAtlas. The bars are shaded according to visual category and show mean PSC across subjects, and error bars indicating the standard error of the mean. *p < .05, **p < .01, ***p < .001 denote results of paired (within-subject) *t*-tests of selectivity for the preferred visual category for each method and for each fROI. (**b**) Each panel corresponds to the remaining 14 fROIs. p-values are listed in Supplementary Table S1.

The critical analysis for evaluating fROIs predicted from connectivity is to confirm if the predicted fROIs are selective for their expected category (e.g., the percent signal change (PSC) for faces in the rFFA is higher than the PSC for all other categories). Figure 2a displays the group means of the actual and predicted PSCs for each category in four key fROIs: the rFFA (selective for faces), the rEBA (selective for bodies), the rPPA (selective for scenes), and the rLO (selective for objects). In the actual and in the predicted rEBA, rFFA, rPPA, and rLO, PSC responses to each preferred category were higher than that for all other categories (all p < 0.001), including objects (which is commonly the contrast category for EBA, FFA, and PPA) and scrambled objects (commonly the contrast category for LO).

**Figure 3:**
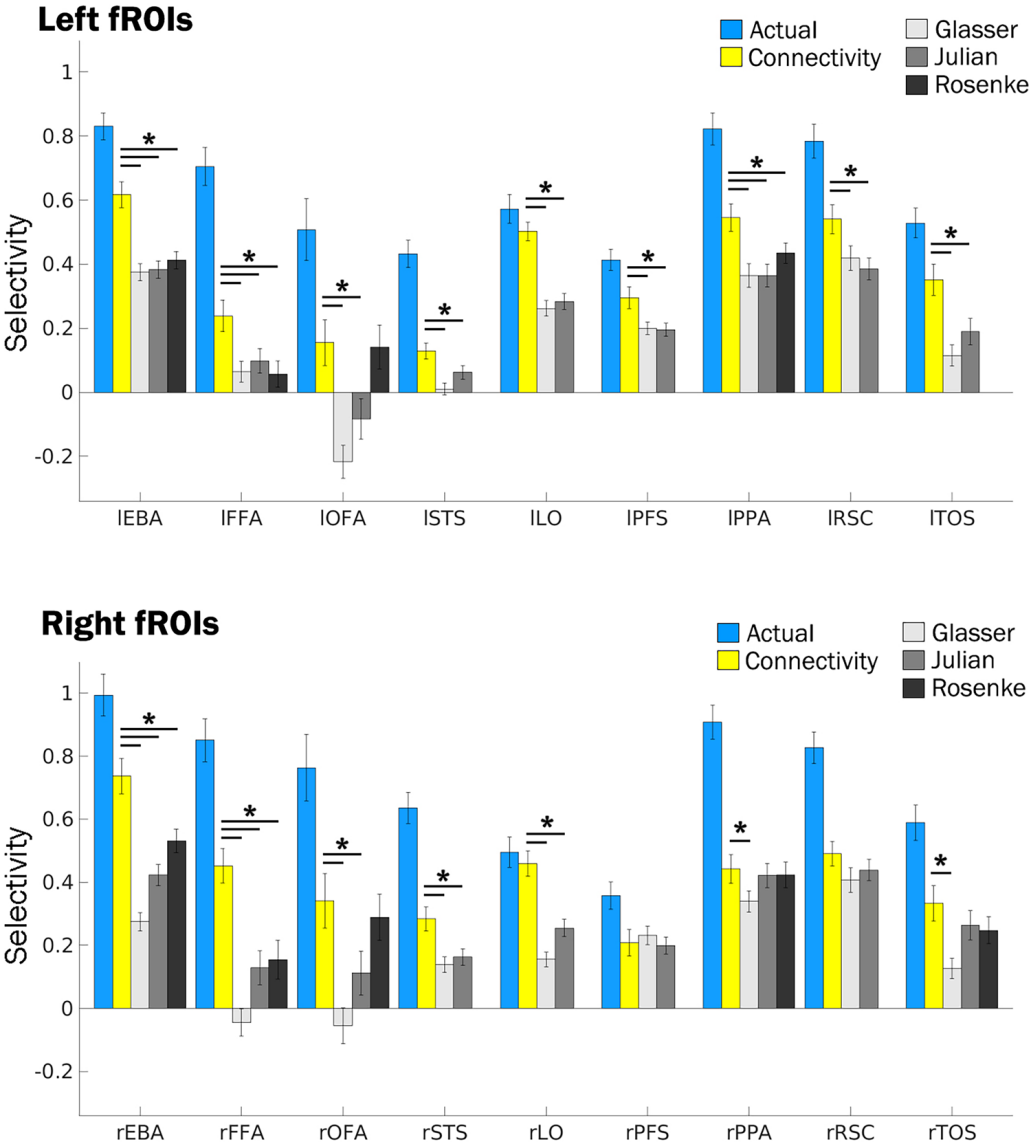
Selectivity for all fROIs. Bar plots showing the mean selectivity for all left (top) and right (bottom) fROIs. The different colored bars indicate the method used to define the region, from left to right: the actual data, the connectivity model, Glasser, Julian, and Rosenke atlases. The lines above the bar indicate a significant difference (Bonferroni corrected for multiple comparisons at p < 0.05) between the connectivity-predicted fROI and each of the atlas methods.

Figure 3 shows the average selectivity for the preferred category of every fROI. As expected, in the actual fMRI data, all fROIs were significantly selective for their preferred category (all p < 5.6 × 10^–6^). Note that in all cases, the fROIs are all most selective when defined from a localizer task, even when defined from only a single run (all p < 5.49 × 10^–4^, Supplementary Table S3). For the connectivity-predicted fROIs, 17/18 regions were significantly selective after accounting for multiple comparisons (Bonferroni-corrected p-value of 0.05/18 regions = 0.00278, see Supplementary Table S1 for raw p-values). The selective regions included: the bilateral EBA, FFA, STS, LO, PFS, PPA, RSC, and TOS (all corrected p < 0.001) and the rOFA (p < 0.01). The lOFA was the only fROI that was not significantly selective after correcting for multiple comparisons (uncorrected p-value = 0.035). PSC plots showing all categories remaining 14 fROIs are shown in Fig. 2b.

### Connectivity fROIs outperform group-level atlas approaches

Next, we compared the selectivity of the predicted fROIs to group-level probabilistic (atlas) parcels. Often, in the absence of task data, researchers define ROIs using parcels delineating the most likely area for a given region based on task data from groups of individuals (such as the search spaces used here from Julian et al.^11^), parcels selected from a general atlas (such as the multimodal parcellation from Glasser et al.^18^), or parcels from an atlas specialized for the domain of interest (such as the visfAtlas of visual regions from Rosenke et al.^19^). The remaining groups of bars in Fig. 2 show the PSC of each category for each of these three methods, referred to as Julian, Glasser, and Rosenke. In the rFFA, for all three methods, the face category does not have the highest PSC, with larger responses to bodies or objects. The rFFAs defined by the Julian and Rosenke methods preferred faces, but were not significantly selective after correcting for multiple comparisons (uncorrected p: Julian = 0.0219, Rosenke = 0.0160). The Glasser parcel actually had a higher mean PSC for objects than for faces, the opposite pattern of what was expected. When testing for significant selectivity (i.e., within individual) in the rFFA, the mean difference between faces and all other categories remained negative. This means on average, the region corresponding to the rFFA had higher PSC in the non-preferred conditions, but this effect was not significant (*p* = 0.31). All atlas methods yielded rPPA, rEBA, and rLO regions that were selective for scenes, bodies, and objects respectively.

Looking across all regions, no method yielded as many significantly selective fROIs as the predictive connectivity model (Fig. 3, see Supplementary Table S1 for exact p-values). For each method: 13/18 Julian parcels were selective for the expected category, 14/18 Glasser parcels were selective, and 6/9 Rosenke parcels were selective. None of these methods yielded a right or a left FFA that was significantly selective for faces. The Glasser and Julian parcels also did not yield a rOFA or lSTS that was significantly selective for faces. Finally, the Julian and Rosenke parcels did not have a selective lOFA. Note that the Julian parcels did not contain any selective fROIs for faces. In all methods, all of the non-selective regions had the preferred category of faces.

Looking within individuals, we next directly compared selectivity within each connectivity fROI to the selectivity within the corresponding fROI defined from the atlas methods (Supplementary Table S2). There were no cases where the Julian, Glasser, or Rosenke parcels had significantly higher selectivity than the connectivity-predicted fROIs. 14/18 connectivity fROIs were more selective than Julian ROIs, with the exception of the rPFS, rPPA, rRSC, and rTOS. Next, 16/18 connectivity fROIs were more selective than Glasser ROIs, except for the rPFS and rRSC. Finally, for the Rosenke parcels, 5/9 connectivity fROIs were more selective, apart from the bilateral OFA, rPPA, and rTOS. In all other regions, the connectivity-predicted fROIs were about as selective as the atlas ROIs.

It is worth noting that comparing the Julian parcels with the connectivity-based fROIs is complicated by the fact that the connectivity-based fROIs are strictly subsets of the Julian parcels. The connectivity models could nonetheless perform either better or worse than the Julian parcels, and here we show that the connectivity-based fROIs tend to be more selective.

### Selectivity of connectivity fROIs varies by region and domain

**Figure 4:**
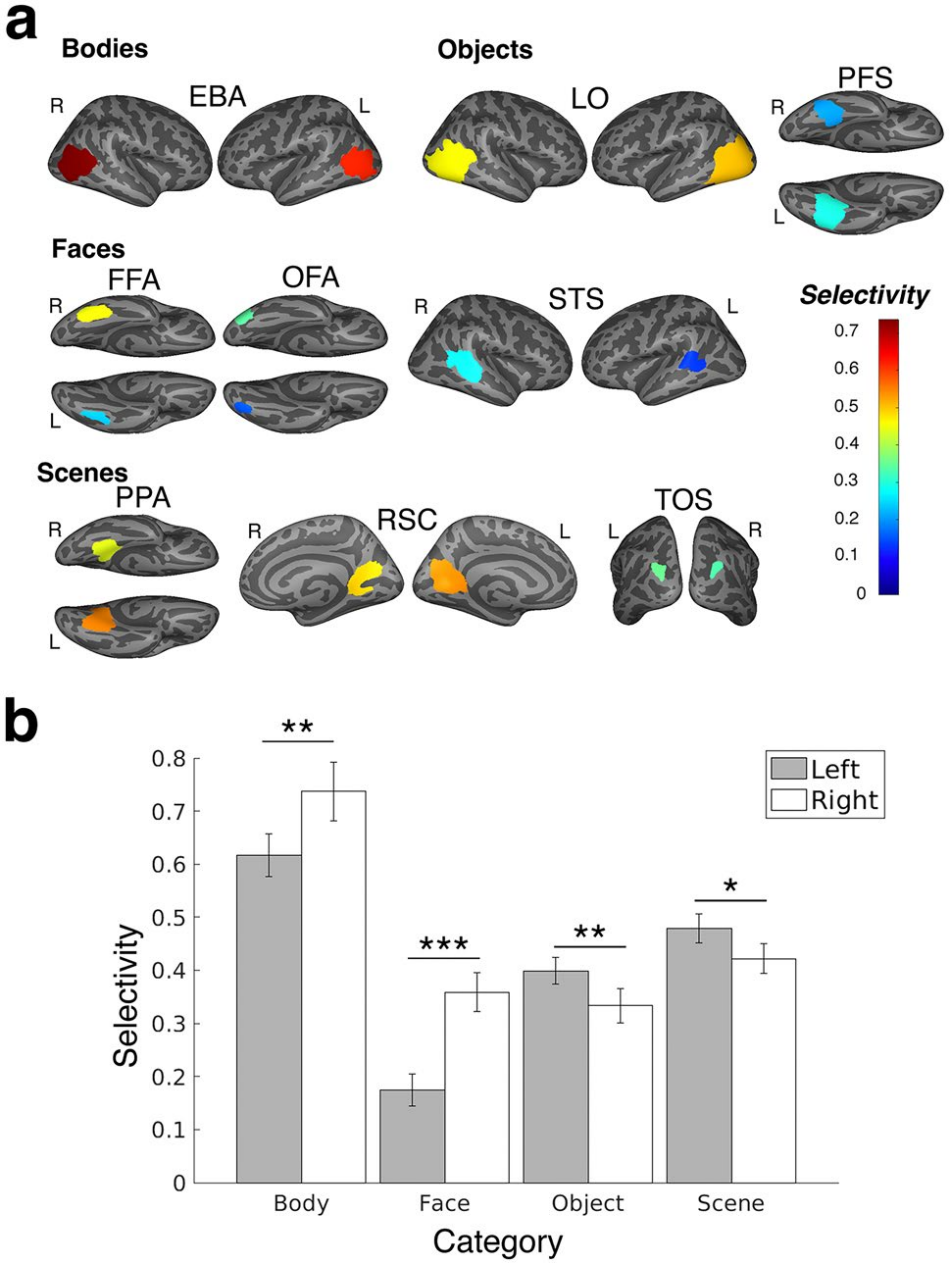
Region and domain differences in selectivity of connectivity-predicted fROIs. (**a**). Each fROI (illustrated as its search space on the surface of the brain) is color-coded by its selectivity. Hotter red–orange colors denote higher selectivity and cooler blue colors denote lower selectivity. (**b**) Mean selectivity averaged across category and hemisphere (gray bar is left hemisphere and white bar is right hemisphere). Error bars denote the standard error of the mean. Bonferroni-corrected ***p < 0.001, **p < 0.01, *p < 0.05.

We next explored how the structure–function relationship varied by fROI, category, and hemisphere. Figure 4a shows the selectivity for each fROI predicted from connectivity plotted on the surface of the brain. The fROIs with the lowest predicted selectivity were two left face regions (lSTS mean: 0.28, lOFA: 0.15), whereas the bilateral EBA had the highest mean selectivity (lEBA: 0.62, rEBA: 0.74). There was a positive relationship between the number of vertices in an fROI’s search space and selectivity of predicted fROIs (Figure S4, r = 0.52, *p* = 0.0279); fROIs defined from larger search spaces tended to be more selective. Averaging within each visual category: predicted body regions were the most selective (mean: 0.68), followed by scene regions (0.45), object regions (0.37), and face regions (0.27).

Finally, we explored if selectivity varied for visual category and/or hemisphere while accounting for individual differences. To quantify this, we conducted an ANOVA of the effect of category and hemisphere on the connectivity-predicted neural activation (main and interaction effects) and subject as a random factor (results in Table 1). Reflecting the group-level results shown in Fig. 3, there were main effects of category (F(3,517) = 13.81, *p* = 9 × 10^–8^) and hemisphere (F(1,517) = 8.17, *p* = 0.0057). Further, there was an interaction effect between domain and hemisphere (F(3,517) = 13.78, *p* = 1.17 × 10^–8^). Post-hoc paired *t*-tests (Fig. 4b, Table S4) revealed the left hemispheric scene and object regions had significantly higher selectivity than the right hemispheric scene and object regions, respectively (scene: t(119) = 2.46, *p* = 0.015, object: t(79) = 3.28, *p* = 0.0015); the right hemispheric face and body regions showed significantly higher selectivity than the left hemispheric face regions (face: t(119) = − 6.12, *p* = 1.25 × 10^–8^, body: t(39) = − 3.38, *p* = 0.0017). This hemispheric difference was most pronounced in the face regions.

**Table 1:** ANOVA Results.

Predictor	Sum of Squares	*df*	Mean Square	*F*	*p*
Domain	11.20	3	3.73	13.81	9.00 × 10^–8^
Hemisphere	0.31	1	0.31	7.85	6.51 × 10^–3^
Subject	6.60	39	0.17	0.77	0.83
Domain*Hemisphere	2.40	3	0.80	13.63	1.43 × 10^–8^
Domain*Subject	31.62	117	0.27	4.61	8.20 × 10^–34^
Hemisphere*Subject	1.36	39	0.03	0.60	0.98
Error	30.28	517	0.06		
Total	85.74	719			

Constrained (Type III) sum of squares.

## Discussion

Here, we computed personalized fROIs responsive to faces, bodies, scenes, and objects, using an individual’s resting state functional connectivity. These regions were selective for their expected category, and overall outperformed group-level parcels, but not fROIs defined from a task-based localizer. Individual differences are known to exist in fROIs, and we found the connectivity predicted regions allow for these individual differences which may be obfuscated in a group-level parcel. Building upon work predicting task activity from connectivity, we demonstrate the feasibility of using fROIs computed from connectivity in place of missing functional task data. We find that resting state data can capture individual variation for high-level visual processes and can be reliably used for defining these fROIs when localizer data is not available or feasible to collect.

In addition to the practical advances of this approach, these results suggest region-to-region variability in the strength of the relationship between connectivity and function. One potential explanation for this variability is the size of the regions, as there was a positive relationship between the size of the search space and selectivity (Supplementary Figure S4). In fact, the only region predicted by the connectivity model that was not significantly selective (the lOFA) had the smallest search space. If there is high individual variability in the location of a putative fROI, then the search space would be large, as the search spaces map out where a given region should be in most individuals. Because the model captures individual variability, it could be the case that the predictions are more reliable in regions that vary greatly from individual to individual. Alternatively, regions with less individual variability in spatial location may be more closely related to cytoarchitecture than connectivity, whereas the locations of regions with large intersubject variability may be driven by individual differences in connectivity. However, it is difficult to determine whether these regions are large because of individual variability, or if the putative fROI itself is large.

The variability in the selectivity of connectivity-predicted fROIs may also reflect variability in the mechanisms underlying visual processing and development of these regions. For example, we observed left/right hemispheric differences in each domain. The most pronounced differences were observed in the face fROIs reflecting differences in hemispheric dominance underlying face (right hemispheric bias) processing^38,39^. Alternatively, regions that are better predicted may have an innately stronger structure/ function relationship. For example, it is known that the neonatal temporal cortex contains connectivity patterns resembling those found in adults^40,41^, but there is evidence some connectivity profiles of regions specialized for some domains (e.g., places) may be more affected by experience than other regions^40^. The observed variability by category (e.g., with the connectivity-predicted face regions having the lowest selectivity) could mirror relatively delayed development of face areas compared to areas specialized for other categories^42^. While our regional differences resemble some of these findings in the broader literature, inferences about the underlying mechanisms and development of these regions are purely speculative.

There are remaining theoretical and methodological questions about predicting high-level visual fROIs. First, in all cases it is preferable to run a task-based localizer, which defines fROIs more precisely than any available method that predicts their locations. It is also worth noting that here a single run of the task localizer was used to define the fROIs^43,44^, and more data, i.e. additional localizer runs, ideally across multiple visits, will yield more accurate individual fROIs^11,45,46^. However, in some cases collecting runs of a localizer task is difficult and/or usable data may be limited, such as in infants and young children^47,48^. Moreover, existing largescale neuroimaging datasets may have collected resting state, but no task data (such as the Autism Brain Imaging Data Exchange, or ABIDE, samples^49,50^), while others (such as the UK Biobank^51^ and the Adolescent Brain Cognitive Development, or ABCD, study^52^) may contain tasks that can localize some but not all of these visual categories, not containing stimuli for e.g., bodies, objects, and/or scenes. We argue that the models developed here would be most useful in cases where localizer tasks are either unfeasible, non-existent, or insufficient.

Second, additional participants from other scanning locations, machine types, and preprocessing steps could better generalize the model to out of sample data, as it is presently unclear how scanner and processing differences may affect predictions. Longer localizer task durations for training the model, or longer resting state scans, such as those utilized in Human Connectome Project, would likely improve our models. Additionally, multiple localizer tasks exist for mapping high-level visual regions, and some conditions (e.g., static versus dynamic stimuli) may be better suited for identifying some regions. Third, this study focuses on functional connectivity from resting state fMRI, but it is unclear if task-based functional connectivity could improve these predictions. Fourth, a linear model was chosen here following similar approaches that successfully predicted visual areas from similar models^23,24^, but a nonlinear model may yield more accurate predictions^33^. Fifth, this model shows evidence of a linear relationship between connectivity and function, but it cannot tell us anything about the causal nature of this relationship. However, evidence suggests that innate connectivity drives the development of these visual areas^53,54^.

Our focus for these analyses was on functional connectivity, due to its link with individual differences and extensive work showing a close correspondence between functional connectivity and task based neural activation. Future work can explore if these regions’ selectivity could improve by including different connectivity measures or adopting another modeling framework or algorithm. Previous work has shown structural connectivity is predictive of neural responses in the high-level visual categories explored here, but fROIs and/or selectivity were not computed in those approaches^23,24^. Further, recent work has shown that structural connectivity is predictive of neural reading-related responses and can predict the location of the visual word form area^55^, a region not investigated in this present study. Additionally, here we use the most common definition of function connectivity (correlation between time series), but other metrics, such as dynamic functional connectivity^56^, contain different information about covariation in resting state fMRI and would likely lead to different results. Lastly, recent work has contended that cortical geometry (a neural field theory model of connectivity based on only the distance between two points on the cortical surface) is more informative of resting state and task-based neural activation than (structural) connectivity^57^. Future work could compare the selectivity of fROIs predicted from these different models of connectivity, from structural connectivity to wave models.

Finally, it is unclear if this model would be appropriate for other age groups. Some high-level visual areas appear to be present very early in life, supported by fMRI studies of visual processing in infancy^47,48^. However, these responses are not yet adultlike, and other visual regions do not emerge until after significant experience (e.g., the VWFA and reading). Additionally, connectivity is known to follow characteristic changes throughout development^58^, so it is unlikely a model based on adult connectivity would predict fROIs as selective as those observed here. Finally, this modeling framework could be extended to predict fROIs in other domains, such as language, social processing, and reading. For these domains, different localizer tasks are used, but a major potential advantage of the approach we show here is that a single resting state scan could predict an entire suite of fROIs across domains that would usually require many additional domain-specific functional runs. Because resting state functional connectivity can predict activity in a variety of tasks^28,29^, this suggests that these predictive modeling approaches can produce selective fROIs in domains beyond high-level vision.

### Supplementary Information

Supplementary Information.

## Data Availability

The data used in this study are available from the corresponding author upon reasonable request. The final model trained on all 40 subjects is available at https://github.com/CognitionBrainCircuitryLab/Predict_fROIs.
