# Supplementary material for: Predicting high-level visual areas in the absence of task fMRI
Source: Sci Rep. 2024 May 18;14:11376. doi: 10.1038/s41598-024-62098-9 (PMC11102456; doi:10.1038/s41598-024-62098-9)
Supplement: Supplementary file 1 — Supplementary Information. [file 41598_2024_62098_MOESM1_ESM.docx]

***Supplementary Materials for* Predicting High-level Visual Areas in the Absence of Task fMRI**

**Table S1**

*Significant Selectivity of fROIs Defined from each Method.*

| fROI | Actual | | Connectivity | | Glasser | | Julian | | Rosenke | |
| --- | --- | --- | --- | --- | --- | --- | --- | --- | --- | --- |
|  | p-value | t-statistic | p-value | t-statistic | p-value | t-statistic | p-value | t-statistic | p-value | t-statistic |
| leba | 7.22x10^-22^ | 19.69 | 3.87x10^-18^ | 15.37 | 5.81x10^-17^ | 14.16 | 6.72x10^-17^ | 14.10 | 4.79x10^-18^ | 15.27 |
| reba | 1.13x10^-17^ | 14.88 | 5.61x10^-16^ | 13.21 | 1.21x10^-11^ | 9.46 | 4.12x10^-15^ | 12.40 | 6.44x10^-17^ | 14.12 |
| lffa | 1.98x10^-14^ | 11.79 | 2.29x10^-5^ | 4.81 | 0.0558 | 1.97 | 0.0155 | 2.53 | 0.181 | 1.36 |
| rffa | 2.66x10^-15^ | 12.58 | 4.08x10^-10^ | 8.28 | 0.311 | -1.03 | 0.0219 | 2.39 | 0.0160 | 2.52 |
| lofa | 5.62x10^-6^ | 5.25 | 0.0351 | 2.18 | 1.40x10^-4^ | -4.22 | 0.198 | -1.31 | 0.0474 | 2.05 |
| rofa | 8.83x10^-9^ | 7.28 | 3.39x10^-4^ | 3.93 | 0.338 | -0.97 | 0.119 | 1.59 | 3.08x10^-4^ | 3.96 |
| lsts | 1.91x10^-12^ | 10.10 | 7.13x10^-6^ | 5.18 | 0.574 | 0.57 | 0.0056 | 2.93 | — | — |
| rsts | 1.30x10^-15^ | 12.86 | 5.19x10^-9^ | 7.45 | 2.18x10^-6^ | 5.55 | 2.34x10^-7^ | 6.25 | — | — |
| llo | 1.78x10^-15^ | 12.74 | 7.89x10^-20^ | 17.23 | 3.43x10^-13^ | 10.72 | 5.42x10^-14^ | 11.41 | — | — |
| rlo | 7.13x10^-13^ | 10.46 | 5.64x10^-14^ | 11.39 | 3.94x10^-8^ | 6.81 | 2.11x10^-11^ | 9.27 | — | — |
| lpfs | 4.44x10^-15^ | 12.37 | 1.26x10^-10^ | 8.66 | 1.93x10^-12^ | 10.10 | 1.42x10^-11^ | 9.40 | — | — |
| rpfs | 4.44x10^-10^ | 8.25 | 1.57x10^-5^ | 4.93 | 1.32x10^-9^ | 7.89 | 8.38x10^-9^ | 7.30 | — | — |
| lppa | 3.79x10^-19^ | 16.46 | 1.15x10^-15^ | 12.91 | 3.70x10^-12^ | 9.87 | 9.63x10^-13^ | 10.35 | 1.98x10^-16^ | 13.64 |
| rppa | 1.32x10^-19^ | 16.97 | 7.67x10^-12^ | 9.62 | 2.57x10^-12^ | 10.00 | 1.23x10^-13^ | 11.10 | 9.52x10^-13^ | 10.35 |
| lrsc | 1.54x10^-17^ | 14.75 | 1.05x10^-14^ | 12.04 | 2.02x10^-13^ | 10.92 | 9.52x10^-14^ | 11.19 | — | — |
| rrsc | 3.51x10^-19^ | 16.50 | 3.97x10^-15^ | 12.42 | 7.66x10^-13^ | 10.43 | 5.96x10^-16^ | 13.18 | — | — |
| ltos | 6.65x10^-14^ | 11.33 | 8.34x10^-9^ | 7.30 | 0.0015 | 3.42 | 4.38x10^-5^ | 4.60 | — | — |
| rtos | 5.67x10^-13^ | 10.54 | 6.46x10^-7^ | 5.93 | 2.79x10^-4^ | 3.99 | 1.31x10^-6^ | 5.71 | 7.33x10^-7^ | 5.89 |

*Note.* p-value from 2-sided paired t-test of an individual’s mean PSC for the preferred category > that individual’s PSC for the contrast category.

**Table S2**

*Comparison of Connectivity fROI’s to Group-level Atlas Approaches*

| fROI | Connectivity vs. Glasser | | Connectivity vs. Julian | | Connectivity vs. Rosenke | |
| --- | --- | --- | --- | --- | --- | --- |
|  | p-value | t-statistic | p-value | t-statistic | p-value | t-statistic |
| leba | 2.55x10^-10^ | 8.43 | 4.82x10^-10^ | 8.22 | 7.85x10^-9^ | 7.32 |
| reba | 4.34x10^-14^ | 11.49 | 2.23x10^-10^ | 8.47 | 4.98x10^-6^ | 5.29 |
| lffa | 2.56x10^-5^ | 4.77 | 0.000243 | 4.04 | 9.97x10^-5^ | 4.33 |
| rffa | 1.35x10^-16^ | 13.81 | 1.36x10^-11^ | 9.42 | 3.69x10^-9^ | 7.56 |
| lofa | 1.31x10^-8^ | 7.16 | 1.34x10^-6^ | 5.70 | *0.706* | 0.38 |
| rofa | 1.75x10^-8^ | 7.06 | 1.72x10^-5^ | 4.90 | *0.184* | 1.35 |
| lsts | 1.82x10^-7^ | 6.33 | 0.000764 | 3.65 | — | — |
| rsts | 6.15x10^-6^ | 5.22 | 6.20x10^-5^ | 4.49 | — | — |
| llo | 1.46x10^-16^ | 13.77 | 8.11x10^-16^ | 13.06 | — | — |
| rlo | 9.68x10^-14^ | 11.19 | 1.90x10^-10^ | 8.53 | — | — |
| lpfs | 9.99x10^-6^ | 5.07 | 7.20x10^-6^ | 5.18 | — | — |
| rpfs | *0.273* | -1.11 | *0.689* | 0.40 | — | — |
| lppa | 1.17x10^-7^ | 6.47 | 1.08x10^-9^ | 7.96 | 0.000108 | 4.31 |
| rppa | 0.00116 | 3.51 | *0.497* | 0.69 | *0.601* | 0.53 |
| lrsc | 0.000827 | 3.62 | 1.81x10^-6^ | 5.61 | — | — |
| rrsc | *0.0147* | 2.55 | *0.0621* | 1.92 | — | — |
| ltos | 6.74x10^-10^ | 8.11 | 2.09x10^-6^ | 5.56 | — | — |
| rtos | 8.78x10^-7^ | 5.83 | *0.0483* | 2.04 | *0.0313* | 2.23 |

*Note*. Italicized p-values not significant with Bonferroni-corrected cutoff > 0.00278. All mean differences were positive, indicating higher selectivity in the connectivity predicted fROIs compared to each method.

**Table S3**

*Comparison of Actual fROIs to Connectivity fROIs*

| fROI | Actual vs. Connectivity | |
| --- | --- | --- |
|  | p-value | t-statistic |
| leba | 4.68x10^-9^ | 7.48 |
| lffa | 1.09x10^-12^ | 10.30 |
| lofa | 3.63x10^-9^ | 7.57 |
| lsts | 1.89x10^-11^ | 9.31 |
| llo | 2.06x10^-5^ | 4.84 |
| lpfs | 8.32x10^-10^ | 8.04 |
| lppa | 3.63x10^-9^ | 7.57 |
| lrsc | 1.00x10^-6^ | 5.79 |
| ltos | 4.39x10^-7^ | 6.05 |
| reba | 4.32x10^-7^ | 6.06 |
| rffa | 1.07x10^-9^ | 7.96 |
| rofa | 9.78x10^-10^ | 7.99 |
| rsts | 2.01x10^-11^ | 9.28 |
| rlo | 5.29x10^-4^ | 3.78 |
| rpfs | 1.57x10^-9^ | 7.84 |
| rppa | 1.47x10^-16^ | 13.77 |
| rrsc | 6.75x10^-9^ | 7.37 |
| rtos | 8.00x10^-8^ | 6.58 |

*Note*. All mean differences were positive and significant, indicating higher selectivity in the actual fROIs defined with an independent run of task data compared to the fROIs predicted by connectivity.

**Table S4**

*Post-hoc Hemisphere x Category Tests*

| Category | Mean Difference (Left-Right) | t | p |
| --- | --- | --- | --- |
| Body | -0.12 | t(39)= -3.38 | 0.0017 |
| Face | -0.18 | t(119)= -6.12 | 1.25x10^-8^ |
| Object | 0.07 | t(79)= 3.28 | 0.0015 |
| Scene | 0.06 | t(119)= 2.46 | 0.015 |

*Note*. 2-sided paired t-test. Note mean difference is the mean selectivity within the connectivity fROIs on the left hemisphere minus the mean selectivity within the connectivity fROIs on the right hemisphere. Negative differences indicate a right hemispheric dominance, while positive differences indicate a left hemispheric dominance.


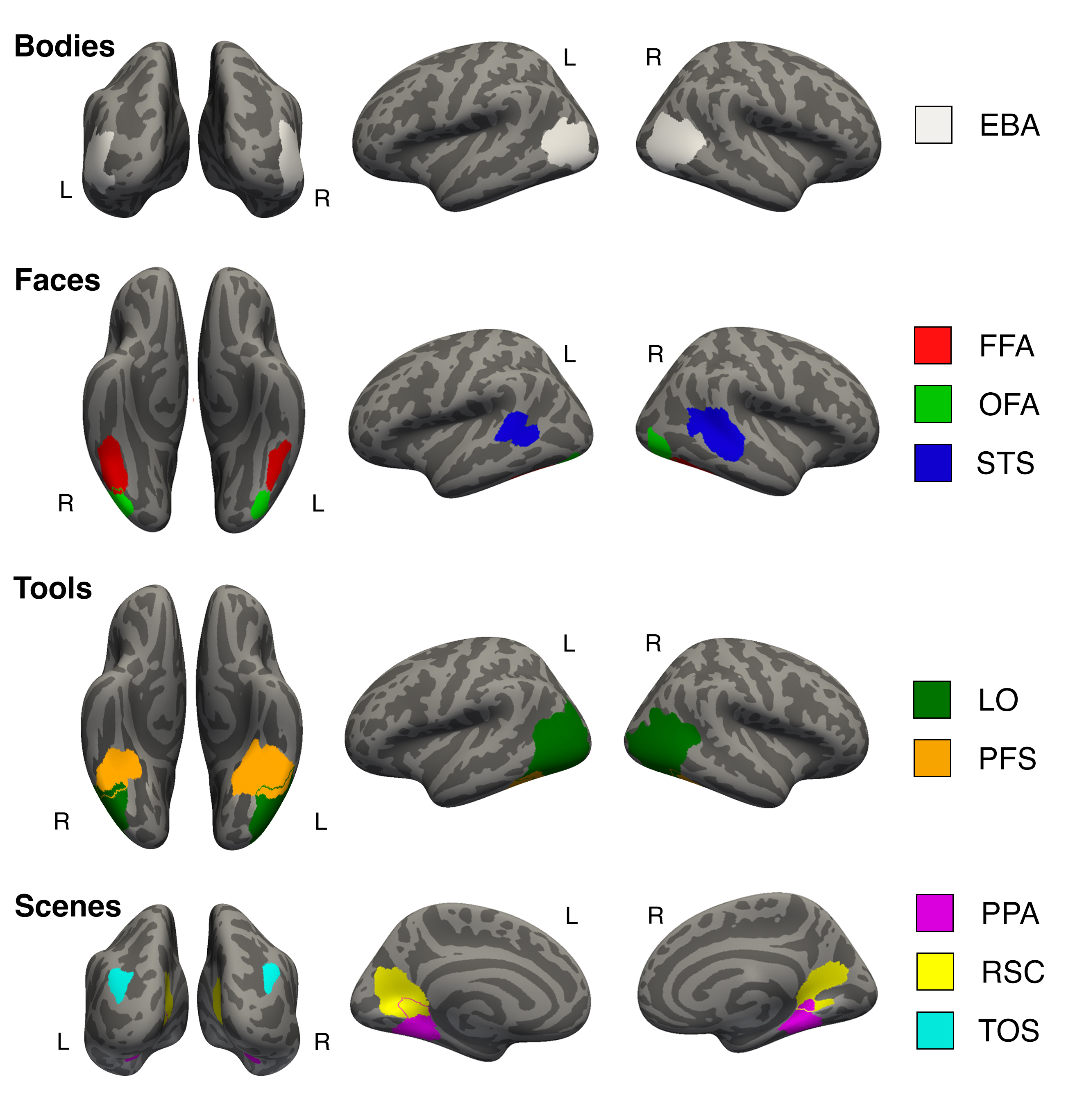


*Figure S1.* **High-level Visual Search Spaces.** The bilateral search spaces from (Julian et al., 2012) plotted on the surface of the brain. These regions include: one body region (EBA: extastriate body area), three face regions (FFA: fusiform face area, OFA: occipital face area, STS: superior temporal sulcus), three scene regions (PPA: parahippocampal place area, RSC: retrosplenial cortex, TOS: transverse occipital sulcus), and two object regions (LO: lateral occipital, PFS: posterior fusiform sulcus). The left (l) and right (r) search spaces were modeled separately for each region.


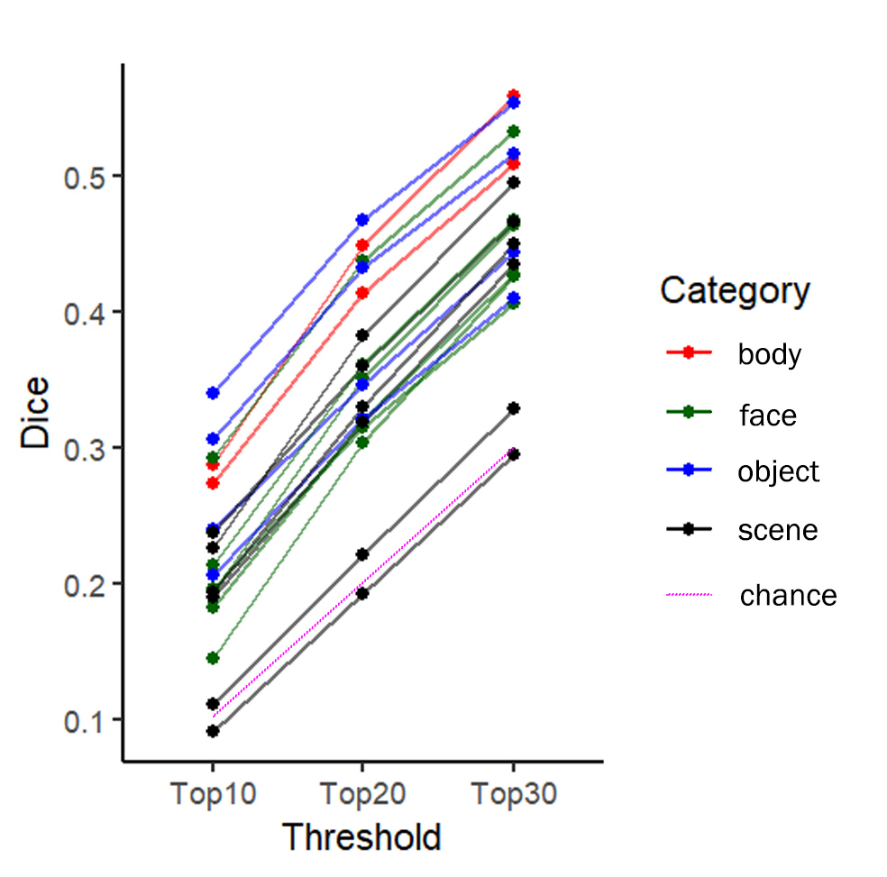


*Figure S2.* **Dice Overlap of Actual and Connectivity Predicted fROIs**. The dice overlap (y-axis) between actual and predicted regions are shown averaged across subjects for each fROI (dots), color-coded by category, when defined by selecting the top 10%, 20%, or 30% of voxels (x-axis). First, at the Top 10% threshold reported in the main paper, we found that the dice overlap between the predicted and actual fROI was on average 0.21966 (minimum average fROI dice was 0.0922, maximum dice was 0.3396), similar to a previously published analysis from Grotheer et al., 2021. The actual fROI overlap, i.e. the dice coefficient between the actual fROI defined from run 1 and the actual fROI defined from run 2 was on average 0.4077, with a minimum dice of 0.2731 and a maximum of 0.5844. We interpret this to mean that on average, we are predicting 53.88% of the explainable overlap in fROI. Second, we found that as the threshold increases, the Dice coefficient also increases, as the regions become larger. However, in all threshold cutoffs, the predicted fROIs performed about the same when accounting for chance (dashed line in plot below). Note one fROI (rPPA) was below chance in each threshold category.


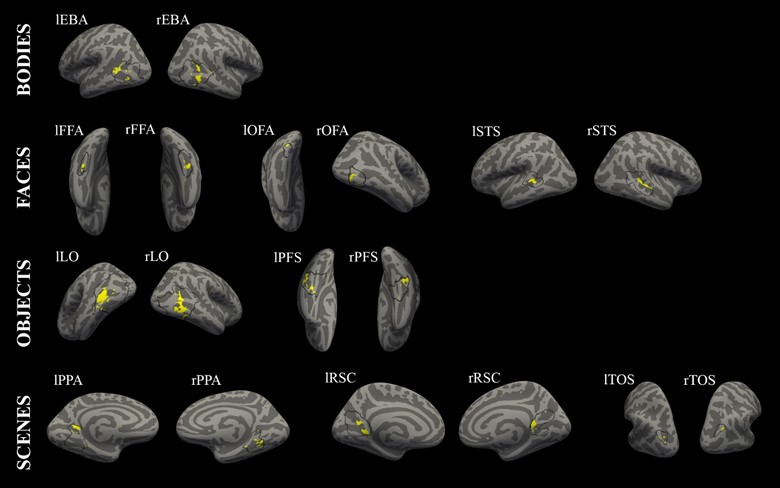


*Figure S3.* **Example fROIs Predicted from Connectivity.** The predicted fROIs are plotted on the surface of the brain in yellow, and the search spaces from Julian et al., (2012) are denoted by the black outlines. Regions include: one body region (EBA: extastriate body area), three face regions (FFA: fusiform face area, OFA: occipital face area, STS: superior temporal sulcus), three scene regions (PPA: parahippocampal place area, RSC: retrosplenial cortex, TOS: transverse occipital sulcus), and two object regions (LO: lateral occipital, PFS: posterior fusiform sulcus). The left (l) and right (r) search spaces were modeled separately for each region.


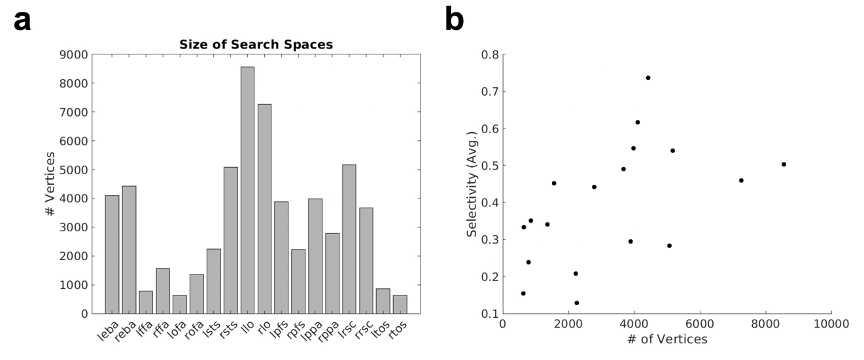


*Figure S4*. **Size of Search Space and Selectivity**. a. Bar plot of the number of vertices for each fROI search space. b. Selectivity vs. size, each point is an fROI.
